# Use of recombinant lentivirus pseudotyped with vesicular stomatitis virus glycoprotein G for efficient generation of human anti-cancer chimeric T cells by transduction of human peripheral blood lymphocytes in vitro

**DOI:** 10.1186/1743-422X-3-8

**Published:** 2006-02-28

**Authors:** Anthony Simmons, Robert P Whitehead, Andrey A Kolokoltsov, Robert A Davey

**Affiliations:** 1 Department of Pediatrics, University of Texas Medical Branch, Galveston, Texas, USA; 2 Department of Internal Medicine, University of Texas Medical Branch, Galveston, Texas, USA; 3 Department of Microbiology & Immunology, University of Texas Medical Branch, Galveston, Texas, USA

## Abstract

**Background:**

Genetic redirection of lymphocytes that have been genetically engineered to recognize antigens other than those originally programmed in their germlines is a potentially powerful tool for immunotherapy of cancers and potentially also of persistent viral infections. The basis for this procedure is that both cancers and some viruses have developed strikingly similar mechanisms of evading attacks by host immune mechanisms. To redirect human peripheral blood lymphocytes (PBLs) with a chimeric T cell receptor (chTCR) so that they recognize a new target requires a high degree of transfection efficiency, a process that is regarded as technically demanding.

**Results:**

Infection with a retroviral vector carrying a chTCR cassette was shown to transduce 100% of rapidly dividing murine T cells but typically, only ~10% of PBLs could be infected with the same vector. In contrast with other retroviruses, lentiviruses integrate their genomes into non-dividing cells. To increase host cell range, vesicular stomatitis virus G protein was pseudotyped with a lentivirus vector, which resulted in ~100% PBL transduction efficiency. Signaling of PBLs bearing chimeric receptors was shown by specific proliferation on exposure to cells expressing cognate ligand. Further, T-bodies against CEA showed a startling abilty to cause regression of maligant colon tumors in a nude mouse model of human cancer.

**Conclusion:**

A lentivirus/VSV pseudotyped virus, which does not require replicating cells for integration of its genome, efficiently transduced a high proportion of human PBLs with chTCRs against CEA. PBLs transduced by infection with a lentivirus/VSV pseudotyped vector were able to proliferate specifically in vitro on exposure to CEA-expressing cells and further they had a startling therapeutic effect in a mouse model of human colon cancer.

## Background

It has become increasingly apparent that the scope of immunization and immunotherapy is applicable not only to infectious agents but also to tumors. Persistent viruses and tumors escape immune surveillance by a variety of common mechanisms, one of the more prominent being down-regulation of class I major histocompatibility molecules thereby preventing recognition by cytotoxic T cells. Antibodies on the other hand retain their ability to recognize native antigens but in case of tumors their potential effects are compromised by their failure to penetrate into neoplastic tissue.

In the current work, the term T-bodies is used to describe T lymphocytes whose targets were redirected using viral vectors engineered to convey to lymphocytes chTCR cassettes, based on a single chain antibody variable fragments for antigen recognition [1-6]. Specifically, the T-bodies described recognize an antigen that is expressed selectively by cells in growing tumors.

Vectors based mainly the on the oncoretrovirus Murine Leukemia Virus (MLV) backbone have been tried by several groups [1-6] to transduce lymphocytes with genes encoding chTCRs to combat experimental tumors in mice with, in practical terms, limited therapeutic success. The major problems encountered, including in our own preliminary experiments, were inability to grow MLV vectors to a sufficiently high titer to infect and therefore transduce a high proportion of PBLs and the dependence on oncoretroviruses on dividing cells in order for integration of their genomes into host cell DNA. Eshhar et al. [7] showed that the efficiency of transduction could be increased to 35–70% by pseudotyping the retrovirus with the envelope protein of another retrovirus, Gibbon ape leukemia virus. In contrast with oncoretroviruses, the genus lentiviruses have the ability to transduce non-dividing cells. In addition to murine killer cells, we describe stable transduction of a very high proportion of human PBLs with a lentivirus/vesicular stomatitis virus pseudotype.

It was shown previously [8] that tissue tropism of lentiviruses can be broadened by constructing pseudotyped viral particles comprising a lentiviral genome enveloped by the surface glycoprotein from vesicular stomatitis virus (VSV-G). VSV, a rhabdovirus, has been commonly used for pseudotyping retroviruses because it is highly stable and confers an exceptionally wide host range, because of the binding of VSV-G to a cell surface lipid. We therefore chose to test the ability of VSV pseudotypted lentivirus containing a chimeric T cell receptor for PBL transduction and compare its efficiency with a well known retrovirus for conveying the same chimeric receptor gene. A models of colon cancer in athymic mice was chosen to explore the efficacy of human T-bodies created by lentiviral vectors against a human tumor. The significance of colon cancer is undisputed. Colorectal cancer is the third most common malignant neoplasm in the world [9] and the second leading cause of cancer deaths (irrespective of gender) in the United States [10,11].

Chemotherapy is commonly used in the treatment both of colon. An alternative approach to chemotherapy that is receiving much attention is adoptive immunotherapy with immune cells that have been manipulated *ex vivo*. Despite its promise, effective responses to adoptive immunotherapy have been documented against only a restricted number of tumor types and this approach to cancer therapy has been further restricted by toxicity associated with the need for exogenous administration of interleukin-2. A prominent reason for failure of both vaccination and adoptive immunotherapy is the common ability of tumors to down-regulate molecules of the major histocompatibility complex, a property also shared with many persistent viruses.

**Figure 1:**
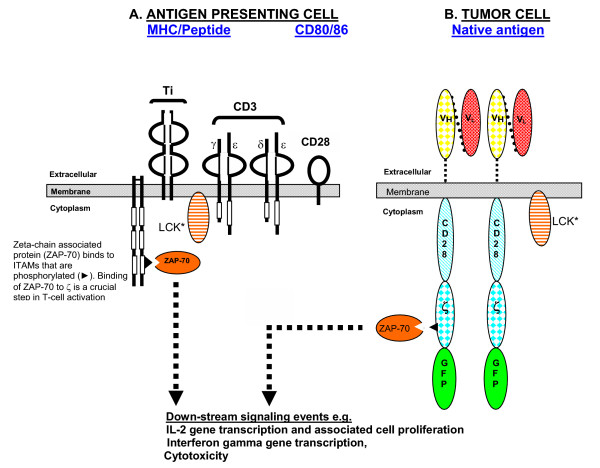
Simplified scheme of T cell activation by A) the T cell receptor and the co-stimulatory receptor, CD28 and B) a chimeric receptor in which co-stimulation and antigen specific activation are provided by a single molecule that is not dependent on MHC-1-associated peptide presentation. *LCK is a lipid raft-associated protein that phosphorylates ITAMs of the TCR zeta chain, allowing them to bind the zeta associated protein kinase ZAP-70. LCK then also activates ZAP-70 by phosphorylation, continuing the T cell signaling cascade. [44]. While it is generally accepted that LCK is activated when CD4 or CD8 co-receptors are cross-linked with the TCR and MHC complex, it is also known that some LCK associates with CD8 in the absence of MHC [45]. It is possible that LCK activation is a function of the surrounding membrane environment rather than the clustering of co-receptors with T cell receptor and MHC [46].

Though it has been established that human tumors may express multiple antigens that can be recognized by cytotoxic T lymphocytes. [12], tumors are not normally attacked by the host's immune system. Binding of antigen to the TCR complex results in a cascade of events commencing with phosphorylation of critical domains of the receptor by membrane-associated Src family of protein tyrosine kinases (PTKs) such as LCK and Fyn, which leads to a cascade of downstream kinase activities that ultimately cause Ca^++ ^fluxing, new gene transcription and/or cell cycle progression (figure 1). A key role in signal transduction is played by domains of the CD3 ζ (zeta) subunit known as Immunoreceptor Tyrosine-based Activation Motifs (ITAMs). When ITAMs are phosphorylated by LYK or Fyn they can bind SH2 domains of other kinases, especially the zeta associated protein, ZAP-70. The ensuing events lead ultimately to transcription of genes, including interleukin-2, that promote cell proliferation.

Conventional wisdom recognizes that activation of naïve T cells requires two signals [13], the primary one being interaction between MHC/peptide and the TCR and a second, co-stimulatory signal, transmitted by interaction between CD28 on the lymphocyte and CD80 (B7-1)/86 (B7-2), generally present on professional antigen presenting cells [14]. Like many T cell receptors, signal transduction by CD28 appears to involve phosphorylation of tyrosine residues in its cytoplasmic domain. The cytoplasmic tail of CD28 is very short (~40 amino acids) and has only four tyrosines, only one of which (tyrosine 188 of the mouse sequence) appears to be essential for co-stimulation as assessed by expression of CD69 and production of interleukin-2 (IL-2). [15].

To feasibly and safely cause regression of carcinomas, the targets recognized by the chTCR is of paramount importance because it must be tumor selective or expressed at very low levels in normal tissues to avoid significant collateral damage by the T-body. CEA was selected for the tumor-selective target in the reported studies because CEA is expressed selectively on most colorectal and several other cancer cell types. As a result it has been a popular target for a variety of immunotherapeutic trials. CEA represents a family of molecules that is involved in regulation of cellular differentiation during embryogenesis. Although it is expressed at very low levels by normal adult tissues, CEA is present in often high levels in cancers of the colon, pancreas, breast, thyroid, lung, ovaries, and stomach.

In clinical trials, colon cancer patients have been vaccinated with CEA peptides, CEA-pulsed dendritic cells or viral vectors containing CEA and co-stimulatory molecules. These strategies occasionally have been shown to engender antigen specific T cell responses and occasionally partial tumor regression in vaccine recipients, which indicates that it may be possible to overcome the potential problems of immune evasion. However, only those patients whose disease is limited to a few specific sites have benefited from this approach to date and moreover the benefits have been short-lived [16-20].

**Figure 2:**
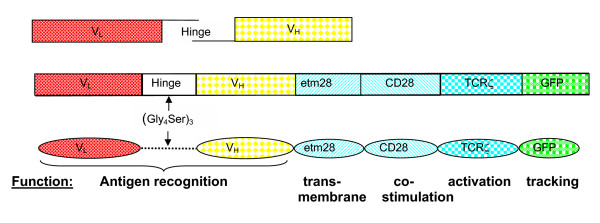
Assembly of a chTCR cassette. Antibody V_L _(red) and V_H _(yellow) were linked by a flexible 15 amino acid hinge to allow correct scFv refolding and reconstitution the antigen binding site. Two thirds of the hinge sequence was attached using specifically designed PCR primers, allowing their splicing to create a scFv. To avoid creation of a sub-optimal (Gly_4_Ser)_2 _hinge, alternative glycine codons were used. To the C terminus of the scFv, extracellular and transmembrane portions of either mouse or human CD28 (etm28) are covalently attached to aid interaction between the chTCR and target and enable fluidity of the chTCR within the cell membrane. The remainder of the chTCR is as described.

Regression of CEA^+ ^colon cancers caused by systemic administration of T-bodies in a scid mouse model of human colon carcinoma was demonstrated previously by Haynes et al [1-3]. Here we show that T-bodies (o2f the structure illustrated in figure 2) constructed from human PBLs by transduction with lentiviral/VSV pseudotypes and directed at CEA have considerable promise for development as therapies for cancer.

## Results

### Redirection of human PBLs and murine MD45 cells with chTCRs by stable transduction

High efficiency stable transduction of lymphocytes is generally regarded as technically demanding. Transfection typically has an unacceptably low efficiency and retroviral transduction is a widely preferred option. This option however requires production of high titer viruses, which are typically packaged in a derivative of NIH3T3 or 293 cells [21].

Direct transfection of PBLs was tried with limited success (<10%) using the calcium phosphate precipitation technique and other contemporary methods (Lipofectamine, Invitrogen; FuGENE 6 and X-tremeGENE, Roche). The focus was then changed to retroviral vectors for introduction of the chTCR cassette into the human genome. Retroviruses have the unique advantage of integration into all host cell genomes and infect a broad range of cell-types. The caveat to the preceding statement is that most retroviruses require rapidly dividing cells to achieve integration but lentiviruses have the capability to integrate into the genomes of non-replicating cells.

**Figure 3:**
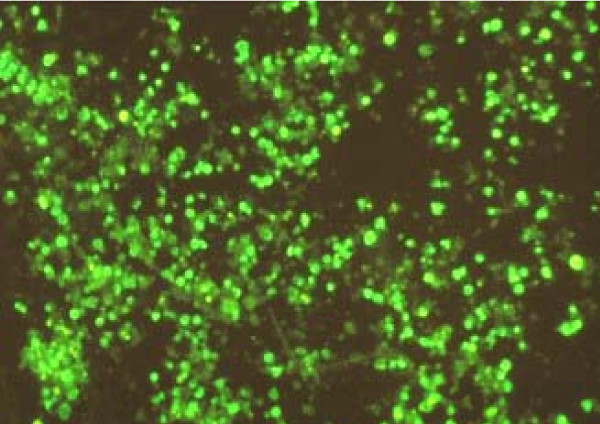
MD45 murine T cell line transduced by infection with a MLV-based retroviral vector (retro-X) containing a GFP- chTCR cassette, demonstrating high efficiency transduction with retro-X and a continuous growing cell population, judged here by expression of GFP.

Whilst high transduction efficiencies could be achieved for a rapidly growing mouse MD45 cell line (Figure 3) using a commercially available MLV-based system (Retro-X, Invitrogen), the maximum viral titer produced by PT67 packaging cells was 10^6 ^transforming units (TU)/ml when titrated in 293 cells. This titer was adequate for infecting all MD45 cells but in contrast, 10^6 ^TU/ml was not sufficient to efficiently transduce human peripheral blood mononuclear cells that were stimulated to proliferate with anti-CD3 and anti-CD28. Typically, only ~10% of cells (not shown) became infected as judged by expression of GFP, a component of the receptor construct. A variety of techniques were tried to increase the viral titer (including overnight incubation of packaging cells at 32°C prior to virus harvest) without additional success. [22].

**Figure 4:**
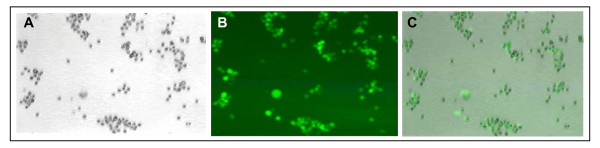
**CEA-specific human T-bodies**. (A) Anti-CD3/CD28 stimulated human PBLs transduced by infection with a VSV/lentivirus pseudotype virus carrying a CEA-specific chTCR (phase); (B) same field viewed by fluorescence for GFP; (C) overlay of A and B, demonstrating the presence of the GFP containing chTCR cassette in all cells.

Consequently, we tried the use of a vesicular stomatitis virus (VSV) G protein pseudotyped lentivirus vector to improve both the viral titer and transduction efficiency of target cells. Using this approach, viral titers of 10^7 ^TU/ml (in 293 cells) or greater were obtained, which enabled 5–10 TU/cell to be used for infection of 10^6 ^PBLs. With this approach it was possible to achieve stable transduction of near 100% of anti-CD3/anti-CD28 stimulated PBLs (Figure 4).

### In Vitro detection of CEA T-body proliferation

**Figure 5:**
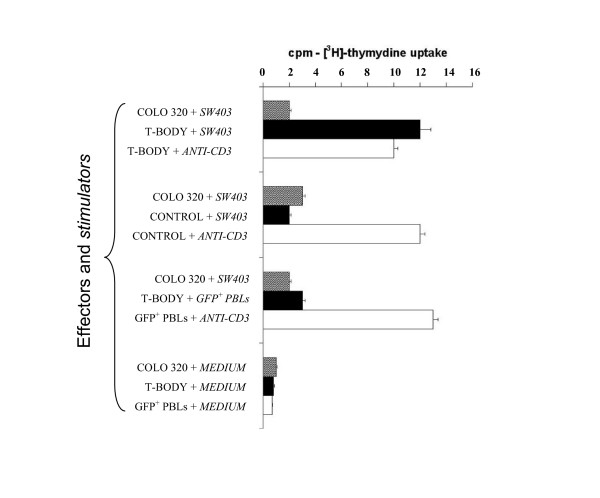
CEA-specific proliferation of human lymphocytes measured by uptake of [^3^H]-thymidine. T-bodies (labeled T-body in the figure) were produced by infection of 10^6 ^PBLs with 10^7 ^TU/ml of a virus comprising a VSV envelope and a lentiviral genome containing the CEA-CD28-CD3zeta-GFP cassette. T-bodies (■) responded to irradiated CEA+ SW 403 stimulator cells but the CEA- COLO 320 control cells () did not. PBLs transduced with a GFP cassette alone did not respond. All cells proliferated vigorously in response to anti-CD3/CD28 (□) and no CEA-CD28- CD3ζ-GFP T-bodies responded to medium alone.

To demonstrate antigen-specific stimulation of human CEA T bodies generated in the laboratory, a standard [^3^H]-thymidine uptake proliferation assay was used (figure 5). PBLs tranduced with the chTCR proliferated vigorously on exposure to irradiated CEA positive (SW403) but not CEA negative (COLO 320 HSR) colon carcinoma cells. Further, little stimulation of control cells tranduced with a GFP cassette alone or untransduced PBLs was observed. Finally, the T-bodies proliferated vigorously on cross-linking of their natural TCRs with anti-CD3 plus anti-CD28. Thymidine uptake in the presence of cell culture medium alone was considered background. The results were interpreted as specific redirected signaling of peripheral blood lymphocytes by the chTCR.

### Regression of tumors in an experimental model of human colon carcinoma

**Figure 6:**
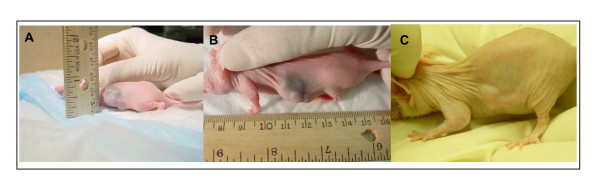
Panels A and B: Established tumor (V = 146 mm^3^) 28 days after subcutaneous injection of COLO320 and SW403 cells respectively into left flanks of NU/J *Foxn1nu *mice (H2^d^). For advanced tumors it was possible to measure W and L using a ruler. A caliper was used for regressing tumors (e.g. panel C, 30 days after intravenous CEA T body treatment; pre- and post -treatment. RTV C vs. B = 0.43).

**Figure 7:**
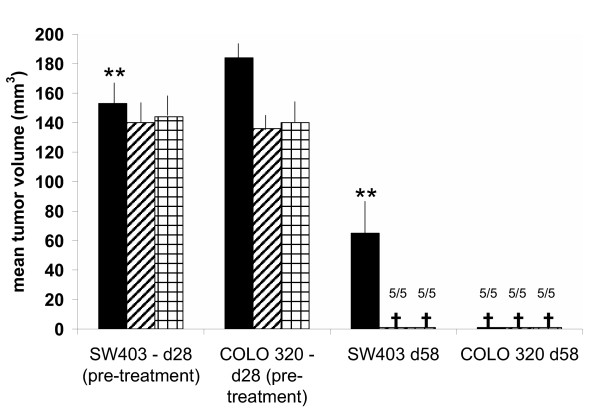
The effect of immunotherapy on the mean volumes (+SEM) of CEA positive and CEA negative human tumors that were established in nude (NU/J *Foxn1nu*) mice (see figure 6). Groups of five mice were injected once with 10^7 ^tumor cells (day 0). On d28 well-established tumors (e.g. see figure 6) were treated by a single intravenous injection of 10^7 ^normal PBLs () or 10^7 ^PBLs transduced using a retroviral vector containing either a CEA-specific scFv-CD28-zeta-GFP chimeric receptor () or a GFP gene alone (). The CEA-specific T-bodies caused unexpectedly startling regression colon tumors 4 weeks after systemic administration of T-bodies (** p < 0.01). Control cells had little effect. The effect was specific for CEA as CEA- (COLO 320) cells were not affected by any cells transferred. By day 58, all mice had died from the tumor (cross) except those treated with the CEA-specific T-body. Despite 100% survival of T-body treated mice on day 58, all groups (treated and untreated) had succumbed to the tumor by 100 days.

Tumor growth was first visible 3 weeks after injection of cells and tumors grew to a diameter of ~1.5 cm 4 weeks after they first became visible (figure 6). 10^7 ^chimeric or control T cells were adoptively transferred via tail veins to tumor cell recipients three weeks after tumor cells. Regression was dramatic (Figure 6 and 7) with CEA-specific T-bodies alone, which are expected to attack only cells at the tumor rim. Experimental colon cancers recurred after treatment with CEA T-bodies and all mice we dead by day 100.

## Discussion

The main thrust of the current work was to show that high efficiency stable transduction of human PBLs is a feasible prospect for generating anti-cancer chimeric T cells for use in cancer immunotherapy. The target selected in the current work was CEA, a tumor-selective antigen

Several possible mechanisms for tumor escape from immune surveillance have been demonstrated in model systems. Mechanisms of immune evasion include presentation of a tumor antigen by tumor cells without the necessary co-stimulatory signal [23,24], suppression or anergy of tumor-infiltrating T-cells [25-29], inability of a tumor antigen to induce high avidity T-cells. [30,31] and possibly most significant and general of all, tumor cells commonly down-regulate expression of either class I or class II major histocompatibility complex (MHC) molecules needed for presentation of antigens to lymphocytes [16,32-34]. Adoptive immunotherapy with autologous chimeric T lymphocytes that recognize a tumor antigen has enormous therapeutic potential to produce regression of tumors in humans with advanced cancers. A useful anti-cancer T-body may be defined as an autologous lymphocyte whose natural target has been redirected to a tumor specific antigen by introduction of a chTCR. The approach that seems most promising in animal models is the use of a single chain antibody variable fragment (scFv) against the tumor coupled to T cell receptor signaling domains for activation effector functions. A great advantage of this approach is that stimulation of a cell with a scFv-based receptor does not depend on expression of major histocompatibility molecules by the target cells. Prior reported studies, using a similar model in scid mice [3] to the nude mouse model described here, demonstrated tumor inhibition when T-bodies were administered on one day after tumor cells.

In the current work, chTCR cassettes combining intracellular CD28 and CD3 sequences were constructed and inserted into lentivirus/VSV pseudotypic vectors which were subsequently used for transduction of PBLs.

A weakness of many prior attempts has been failure to take into account the need for T cells to receive a second, co-stimulatory, signal from accessory molecules on antigen presenting cells such as CD80 and CD86. However, many T bodies have been investigated that supply only a single activation signal, generally from ITAMs derived from the CD3 ζ (zeta) chain component of the T cell receptor. Addition of the part of the small intracellular domain of the co-stimulatory receptor molecule CD28 has been shown to improve the responses of T-bodies in vitro and in vivo. [2]. Recently, Hombach et al. [6] examined the requirement for stimulation of CD28 in chTCR by CD80/86 and found that proliferation, cytokine secretion and cytolysis were differentially modulated by receptor cross-linking. These authors found that cytolysis in particular did not require an interaction between CD28 and CD80/86. The implications of these findings are that, while tumor cell lysis by chimeric T cells is independent of CD28, IL-2 secretion will be lacking under these circumstances. A lack of IL-2 has the obvious consequence of impaired Th1 cellular responses, for which IL-2 is a potent stimulator. Thus there may be deficient recruitment of natural killer and other key effector cells. Pinthus et al. [35] used a strategy of preconditioning the bone marrows of immunodeficient mice to accept redirected effector lymphocytes, by total body irradiation or low doses cyclphosphamide. This had the effect of stimulating secretion of SDF-1, a powerful mediator of chemotaxis for CXCR-4 expressing killer cells, improving the homing efficiency of chimeric PBLs to bone marrow and enabling artificially induced bone metastases from prostate cancer to be treated successfully after intravenous administration of T-bodies. It remains to be shown whether similar strategies will be required to prepare other metastatic sites for retention of adoptively transferred T bodies.

Unlike the signal transduction events that follow ligand binding by the natural TCR, which involve clustering of CD28 (and perhaps other) molecules capable of providing co-stimulation into the same vicinity, the chimeric TCRs generated here provide stimulation and co-stimulation in an antigen-dependent manner from the same molecule. Haynes et al. [2] showed previously that co-stimulation provided superior efficacy over CD3-zeta alone for stimulating chimeric T-cells.

It is possible that chTCR may be recruited to lipid rafts and this may provide an explanation for their ability to transmit a signal to the host cell, given the known association of LCK with rafts. Understanding the molecular processes involved in activation of T cells via a chTCR is important because host T cells may fail to respond to stimuli [36], at least in part, due to abnormal expression of signal transduction molecules [37,38], which may create a barrier to use of chTCR that depends on proximal components of the T cell signaling cascade. Fitzer-Atlas et al. [39] demonstrated that a scFv-PTK chTCR could bypass proximal TCR transduction steps and directly stimulate T cell effector mechanisms, indicating that inclusion of distal members of the TCR stimulation cascade offers alternative approaches to the receptor structure described here.

## Conclusion

We conclude that pseudotyped virus comprising a lentivirus, which does not require replicating host cells for integration of its genome, together with an envelope containing vesicular stomatitis virus glycoprotein G, is superior to an oncoretrovirus carrying the same transgene for efficient transduction of human PBLs. Using this approach we were able to efficiently redirect PBLs with chTCRs against a human tumor selective target. PBLs expressing CEA-specific chimeric receptors proliferated specifically in vitro on exposure to CEA-expressing cells. CEA specific T bodies had startling therapeutic effects in a mouse model of human colon cancer. Thus, this vector has potential for redirection of human PBLs to chimeric anti-tumor T cells, forming basis for immunotherapy of many different human cancers

## Methods

### Mouse cells and human lymphocytes

MD45 cells, a murine cell NK-like T cell line, were obtained from Zelig Eshhar (Weizmann Institute of Science, Rehovot, Israel). PBLs were separated from 100 ml samples of whole blood by centrifugation (800 Xg) through Ficoll-Plaque Plus (Amersham Biosciences). Banded PBLs were washed twice in phosphate buffered saline and resuspended in RPMI1640 (Gibco, NY) at a concentration of 10^7^cells/ml. All cells were propagated in a 5%CO_2 _atmosphere using RPMI1640 (Gibco, NY) supplemented with10% fetal bovine serum, 2 mM L-glutamine, antibiotics (penicillin/streptomycin), 10 mM HEPES Buffer, 10 mM and sodium bicarbonate.

### Generation of chTCRs against CEA

The chTCR against CEA was generated from:

An anti-human CEA single chain antibody which was provided by Hinrich Abken (Cologne, Germany).

CD28 sequences that were PCR cloned in one section from human cDNA prepared by reverse transcription of splenocyte DNA using published primers [40].

### Generation of lentiviral vectors

The expression cassettes described above were inserted into a derivative of pLENTI6 (Invitrogen, CA) that drives transgene expression under control of a CMV promoter. Lentivirus was then produced by calcium phosphate-mediated transient transfection of the lentiviral expression construct together with pLP1 (encodes the HIV gag-pol structural proteins), pLP2 (encodes HIV rev) and pVSV-G (encodes VSV G protein) into 293FT cells. Transfection efficiency of cells was measured by detection of GFP expression in the 293FT cells, 2 days post-transfection, and typically exceeded 95%. At the same time culture supernatants containing lentivirus were harvested and filtered through a 0.45 μm filter to remove cell debris. Virus was concentrated when required by centrifugation for 3 h at 25,000 × g. Pellets were then resuspended in DMEM and used immediately or frozen in aliquots at -80°C.

### In vitro cell proliferation assay

To test the ability of T-bodies constructed in the way described to generate a signal from the chimeric receptor, the ability of CEA-specific cells to proliferate in vitro when stimulated by soluble recombinant CEA (Protein Sciences Corp, Meriden, CT) was examined by measuring uptake of ^3 ^[H]-thymidine. Serial dilutions of each sample were tested in triplicate, starting with 10^6 ^cells/100 μl incubated in Aim-V serum free medium (Invitrogen) in wells of 96-well round-bottom microtiter plates. Cells were stimulated with 10 μg/ml recombinant CEA and unstimulated control cells were also included in the assay. Uptake of ^3 ^[H] was used as a standard measure of proliferation [41]. During the final 16 hours of culture, the cells were pulsed by adding 1 μCi ^3^[H]-thymidine to each well. Cells were harvested and the uptake of isotope was measured standard using a Wallac 1205 Beta plate liquid scintillation system (Wallac Inc., Gaithersburg, MD).

### Induction of human tumors in athymic BALB/c mice

All animal experiments were done in compliance with the Animal Welfare Act (P.L. 89–544, as amended by P.L. 91–579, P.L. 94–279, and P.L. 99–108), The Guide for Care and Use of Laboratory Animals (NIH Publication No. 93-23, 1985 or succeeding revised editions), and the PHS Policy of Humane Care and Use of Laboratory Animals.

To simulate a human colon cancer in vivo, groups of five athymic (NU/J *Foxn1nu*) 'nude' mice (H2^d^) were injected subcutaneously (day 0) with either: 10^7 ^CEA+ (SW403; ATCC CCL-230) colon cancer cell or 10^7 ^CEA- (COLO 320; ATCC CCL 220.1) colon cancer cells.

With both cell-types, tumor growth was first visible 3 weeks after injection of cells and tumors grew to a diameter of ~1.5 cm in 4 weeks (e.g. figure 6).

### Therapy of established tumors with lentiviral transduced T cells

10^7 ^chimeric or control T cells were adoptively transferred via the tail veins to tumor cell recipients three weeks after tumor cells. Prior reported studies using a model of colon cancer in scid mice [3] demonstrated tumor inhibition when T-bodies were administered one day after tumor cells were administered. To assess the impact of therapy on advanced tumors, a conventional approach was used for calculating tumor volumes [42] which involves measuring tumor widths in two perpendicular planes and calculating their volume using the following formula for ellipsoid tumors. [43]:

V = W^2 ^× L × 0.52, *where*

V = volume, W = the largest tumor diameter in centimeters and L = the smallest tumor diameter.

Before and 4 weeks after therapy the individual relative tumor volumes (RTV) were used as an objective measure of efficacy and calculated as follows:

RTV = V2/V1 where V2 is the volume in cubic millimeters 4 weeks after a single intravenous injection of T-bodies and V1 is the volume at before T-body administration.

## Competing interests

The author(s) declare that they have no competing interests.

## Authors' contributions

AS and RPW conceived of this project and coordinated all experiments described. RAD and AK were responsible for advice and assistance with making the lentivirus-VSV pseudotyped viruses.
